# Exploiting in silico modelling to enhance translation of liver cell therapies from bench to bedside

**DOI:** 10.1038/s41536-024-00361-3

**Published:** 2024-05-09

**Authors:** Candice Ashmore-Harris, Evangelia Antonopoulou, Simon M. Finney, Melissa R. Vieira, Matthew G. Hennessy, Andreas Muench, Wei-Yu Lu, Victoria L. Gadd, Alicia J. El Haj, Stuart J. Forbes, Sarah L. Waters

**Affiliations:** 1 Centre for Regenerative Medicine, Institute for Regeneration and Repair, The University of Edinburgh, Edinburgh BioQuarter, 5 Little France Drive, Edinburgh, EH16 4UU UK; 2 Mathematical Institute, University of Oxford, Oxford, OX2 6GG UK; 3 Healthcare Technologies Institute (HTI), Institute of Translational Medicine, University of Birmingham, Birmingham, B15 2TH UK; 4 School of Chemical Engineering, College of Engineering and Physical Sciences, University of Birmingham, Birmingham, B15 2TH UK; 5 Department of Engineering Mathematics, University of Bristol, BS8 1TW Bristol, UK; 6 Centre for Inflammation Research, Institute for Regeneration and Repair, The University of Edinburgh, Edinburgh, EH16 4UU UK

**Keywords:** Therapeutics, Regeneration, Cell delivery

## Abstract

Cell therapies are emerging as promising treatments for a range of liver diseases but translational bottlenecks still remain including: securing and assessing the safe and effective delivery of cells to the disease site; ensuring successful cell engraftment and function; and preventing immunogenic responses. Here we highlight three therapies, each utilising a different cell type, at different stages in their clinical translation journey: transplantation of multipotent mesenchymal stromal/signalling cells, hepatocytes and macrophages. To overcome bottlenecks impeding clinical progression, we advocate for wider use of mechanistic in silico modelling approaches. We discuss how in silico approaches, alongside complementary experimental approaches, can enhance our understanding of the mechanisms underlying successful cell delivery and engraftment. Furthermore, such combined theoretical-experimental approaches can be exploited to develop novel therapies, address safety and efficacy challenges, bridge the gap between in vitro and in vivo model systems, and compensate for the inherent differences between animal model systems and humans. We also highlight how in silico model development can result in fewer and more targeted in vivo experiments, thereby reducing preclinical costs and experimental animal numbers and potentially accelerating translation to the clinic. The development of biologically-accurate in silico models that capture the mechanisms underpinning the behaviour of these complex systems must be reinforced by quantitative methods to assess cell survival post-transplant, and we argue that non-invasive in vivo imaging strategies should be routinely integrated into transplant studies.

## Introduction

### Cell therapies for liver disease

Cirrhosis and other liver diseases were amongst the top five causes of death in 2018 in 20–64 year olds across England, Wales and Scotland, and the leading cause of death for 25-49 year olds in England and Wales^1,2^. For end-stage liver disease, orthotopic liver transplantation (OLT) is the only curative treatment. However, the rising global burden of liver disease means patient demand for liver transplants consistently exceeds the availability of donor organs. In the UK and USA 10-17% of patients each year on the waiting list for a suitable liver transplant die or are moved to palliative care due to their condition deteriorating to the point that they are unlikely to survive a transplant^3,4^. As a result, clinicians and researchers are increasingly considering substitutes for OLT, and cell therapies are proving a promising alternative.

Cell therapies, where viable whole cells are administered to a patient, aim to either cure liver disease or bridge patients to OLT. The therapeutic effect depends on the cell type administered. For hepatocyte transplants (HTx), hepatocytes are isolated from livers rejected for OLT (due to prolonged ischaemia, aberrant anatomy, steatosis, geriatric donors, etc) and infused into patients with liver disease^5^; patients can benefit directly from the metabolic function of the engrafted cells within their damaged tissues. Since the first allogeneic adult HTx was performed in 1997^6^, over 100 patients have been treated^7^ for a range of diseases including metabolic disorders^6,8^, cirrhosis^9^ and acute liver failure (ALF)^6,10,11^. Many patients were successfully bridged to OLT, or fully recovered without requiring OLT^12^. However, graft survival varies between patients and aetiologies, with outcomes often transient (partly attributed to limitations in hepatocyte source material standardisation due to poor donor availability and cell quality). The greatest success of HTx has been found in patients with metabolic disorders, however significant improvements are needed for HTx to successfully treat ALF and cirrhosis. To date no patients have undergone HTx as part of a designated clinical trial, despite several registrations (NCT00282542, NCT01345565, NCT01345578, NCT01465100). This is reportedly due to insufficient finance, potentially because of the transient nature of outcomes registered in case reports.

While the goal of HTx is for administered cells to engraft and repopulate the damaged tissues, for some cell therapies the aim is to facilitate self-healing of damaged tissues. These cell therapies modulate the local immune environment and/or act as functional cells to promote localised tissue regeneration. For example, pre-clinical studies demonstrate that monocyte-derived macrophages promote fibrotic scar resolution through phagocytosis, increased anti-inflammatory cytokine production and promotion of host hepatic progenitor cell proliferation and differentiation^13–16^. The use of autologous monocyte-derived macrophages in a Phase I clinical trial has demonstrated safety in compensated liver cirrhosis patients of differing aetiology, with 6/9 patients showing a reduction of Model for End-Stage Liver Disease (MELD) scores at 90 days post-infusion^17,18^. A phase II trial investigating therapeutic efficacy relative to standard care is in progress (ISRCTN 10368050, EudraCT; reference 2015-000963-15)^19^. Further polarising macrophages into alternatively activated macrophages (AAMs) has been shown in pre-clinical studies to be a potential therapy for acetaminophen (paracetamol, APAP) induced ALF^20^. They are also thought to promote regeneration, through reduction of hepatocellular necrosis, infiltrating neutrophils and circulating pro-inflammatory cytokines, and increased proliferation of native hepatocytes^20^. These promising preclinical results led to a phase I trial investigating therapeutic safety in humans with APAP overdose, which is currently underway (ISRCTN12637839).

Multipotent mesenchymal signalling/stromal cells (MSCs), whilst diverse in tissue of origin and characterisation, are perhaps the most investigated cell therapy for liver disease with a number of case control, cohort studies and clinical trials performed to date (reviewed previously^21,22^). Similarly to macrophage therapies, MSCs are considered immunosuppressive and act in an anti-inflammatory manner, re-polarising tissue resident macrophages and increasing matrix metalloprotease production, which supports resolution of fibrotic scarring^23^. While there is strong interest in these cells, and their safety has been confirmed, not all studies to date have reported efficacy^21^. Given the inconsistent criteria of MSC identity and their inherent heterogeneity, robust randomised trials are still needed for confidence in clinical efficacy ahead of their wider adoption as a potential cell therapy for liver disease.

We acknowledge that many other cells have demonstrated therapeutic potential, including a variety of progenitor cells^24–26^, haematopoietic stem cells^27^ and pluripotent stem cell derived hepatocyte-like-cells^28^, and these have been comprehensively reviewed elsewhere^29,30^. We focused here on MSCs, HTx and macrophages as they are at different stages in their clinical journey: MSCs have undergone several controlled trials to date with varying success, HTx has yet to become a routine therapy despite many clinical case reports and preclinical studies to date, and macrophages are in phase II trials for liver cirrhosis having demonstrated safety in phase I, with Phase I safety studies for acute injury also ongoing^18^. We argue that wider use of mechanistic in silico modelling approaches can help address translational bottlenecks that prevent promising candidate cell therapies from realising their full clinical potential. Major translational bottlenecks include i) ensuring safe and effective delivery of cells to the disease site; ii) enabling sufficient cell engraftment and/or function within the injured niche; and iii) preventing immunogenic responses which could lead to therapeutic rejection. Wider use of real-time in vivo cell tracking technologies during preclinical studies can enhance our understanding of the anatomical distribution (biodistribution) of delivered donor cells, as well as their engraftment within the injured tissue niche. However, the complexity of the liver injury response makes it challenging to identify, using experimental approaches alone, the microenvironmental changes or cellular responses that are key to improving therapeutic interventions due to the significant numbers of animal studies required to identify causal mechanisms using conventional analysis methods. The use of in silico models can advance the fundamental understanding of the mechanisms that govern the patient response to donor cells. Specifically, in silico models can be exploited to compensate for inherent differences between animal model systems and humans, which may currently result in a mismatch between preclinical and clinical outcomes. They can also assist in identifying potential mechanisms to promote injury repair by simulating scenarios which have not been experimentally tested, and these predictions can subsequently be verified by fewer, more targeted experiments. This reinforces the 3Rs principles of replacing, reducing and refining animal experimentation and has the potential to substantially reduce the costs of translating promising cell therapies to the clinic by reducing the number of time consuming and expensive in vivo experiments. To develop validated in silico models, quantitative temporal and spatial experimental data is required to assess the impact of the cell therapies post-delivery, to obtain this data non-invasive imaging strategies must be routinely integrated into transplant studies.

### Mathematical modelling

Here we briefly review how mathematical models can be exploited alongside experimental approaches to advance understanding of complex biological systems involving numerous interactions between components (e.g. cells, extracellular matrix (ECM), interstitial fluid) across spatial and temporal scales. For a comprehensive review of the role of mathematical modelling in the wider regenerative medicine context, we refer the interested reader to Waters, Schumacher and El Haj^31^.

Mathematical models for simulating biological systems can be statistical or mechanistic. While our focus here is predominantly on mechanistic mathematical modelling, we also highlight a statistical modelling approach which has been successfully applied in the clinical setting to islet cell therapies. Statistical models aim to fit or learn the relationship of input variables, such as experimental parameters or biological variables, to output variables, such as experimental measurements. Examples of statistical models are general linear models, logistic regression, and machine-learning techniques such as artificial neural networks.

Mechanistic mathematical model development relies on interrogating experimental observations of the biological system and generating hypotheses for the causal mechanisms underpinning the system behaviour. For example, based on experimental data we can hypothesise how the level of inflammation and scarring in the liver injury microenvironment impacts engraftment of the administered cells. Mathematical representations of the causal mechanisms result in a set of model equations. A range of mechanistic modelling approaches exists, including continuum models which model average cell behaviour by, for example, capturing the cell population via its density that is a continuous function of space and time, and discrete, whereby individual cells are considered via the specification of rules within a computational framework. Hybrid discrete-continuum models integrate both these approaches, for example by explicitly considering how individual cells, modelled as discrete entities, interface with the surrounding ECM, with the equations of continuum mechanics exploited to capture the mechanical stress experienced by the cells embedded within the ECM. A further distinction when considering mechanistic models is the use of deterministic models versus stochastic models. In deterministic models, the model output, e.g. the evolution of cell density over time, is completely specified by the model parameters and initial conditions, and will always return the same solution for a given initial state. In contrast, stochastic models capture the inherent randomness in biological systems. The output of a stochastic simulation is then one realisation of the model behaviour, and the average models behaviour is obtained by performing the stochastic model simulation many times to obtain a full distribution of predictions for the model outcomes. Analytical and numerical tools are then used to determine the range of input-output behaviours predicted by the model.

An important step in the development of mechanistic mathematical models is the identification of model parameters. Such model calibration is performed by quantitatively comparing the theoretical model outputs with available experimental data, and Bayesian parameter inference techniques can be used to solve the inverse problem of which parameter values are most likely to produce the observed experimental data. Once parameterised, the models are validated by testing the predictions against newly generated experimental observations, and discrepancies between model predictions and experimental data then motivate model refinement.

After validation of the mechanistic model with experimental data, model parameters can be varied to mimic conditions for which experimental data has not been generated (e.g. modified levels of inflammation in the system as a result of drug administration). One exciting use of modelling approaches is to bridge the gap between animals and humans by ‘scaling’ in silico models of animal systems to reflect anatomical scales and physiological regimes observed in humans. For example, when considering the transit of cells via the vasculature towards the delivery site, fluid flow in vessels is characterised by the Reynolds number, Re=Ua ρ/μ, where U is the typical velocity, a the vessel radius and ρ and μ are the density and dynamic viscosity of the fluid respectively. The predictions of animal models in terms of advection of the donor cells by the blood flow can then be translated into clinical scenarios via an adjustment of the Reynolds number in the flow calculations. In silico tools can also bridge the gap between in vitro and in vivo models, for example by translating understanding gained from in vitro systems into the in vivo setting through inclusion of additional cell types, e.g. an immune compartment. For example, while cell types of interest can be selected for use in in vitro models, in vivo models include a full immune system compartment. While this is challenging to include all the cellular actors in vitro, in silico models can incorporate and explore the impact of the inclusion of additional cell players, e.g. immune cells such as T cells.

### Paper overview

This paper is organised as follows. We first highlight how existing pre-clinical animal models for liver cell therapies are limited in their ability to predict clinical outcome. We then discuss quantitative experimental methods to assess cell delivery and the impact of donor cells on disease outcome. These methods can be exploited to address safety bottlenecks to cell therapy translation by allowing the biodistribution of cells to be monitored in real-time, and the spatiotemporal data obtained can underpin the development of mechanistic mathematical models. We argue the case for the enormous potential of mathematical models in the field of liver cell therapies by showcasing three examples. The first example is a statistical modelling approach applied clinically to islet cell therapies. The second example is a mechanistic model of hepatitis C virus kinetics and its response to interferon-alpha treatment: it showcases the potential for modelling in hepatology, and the modelling approach is readily translated into cell therapy applications. We end our examples with a recent mathematical model for magnetically targeted stem cell therapies that demonstrates how in silico approaches can be used to bridge the gap between in vitro and in vivo model systems, and provide insights that can be used to overcome safety and efficacy challenges encountered in the development of cell therapeutic approaches. Such an approach has huge potential in the field of liver cell therapies.

## Limitations of preclinical models for cell therapy

We now discuss the challenges encountered in translating liver cell therapies into the clinic, focusing on the limitations of preclinical animal models in predicting the clinical success of a cell therapy. Typically, prior to early stage clinical trials, mouse models are used to determine cell therapy efficacy in vivo. However, even promising cell therapies can show variations in efficacy dependent on the mouse strain. Preparing cells in a GMP-compliant manner and administering them to the ideal mouse model mimicking the phenotype of the intended recipient patient population does not guarantee the same prognosis for future clinical trials. Variations between preclinical and clinical outcomes can be attributed to both inherent anatomical and physiological differences between the model system and humans, as well as unavoidable differences in experimental protocols.

**Fig. 1:**
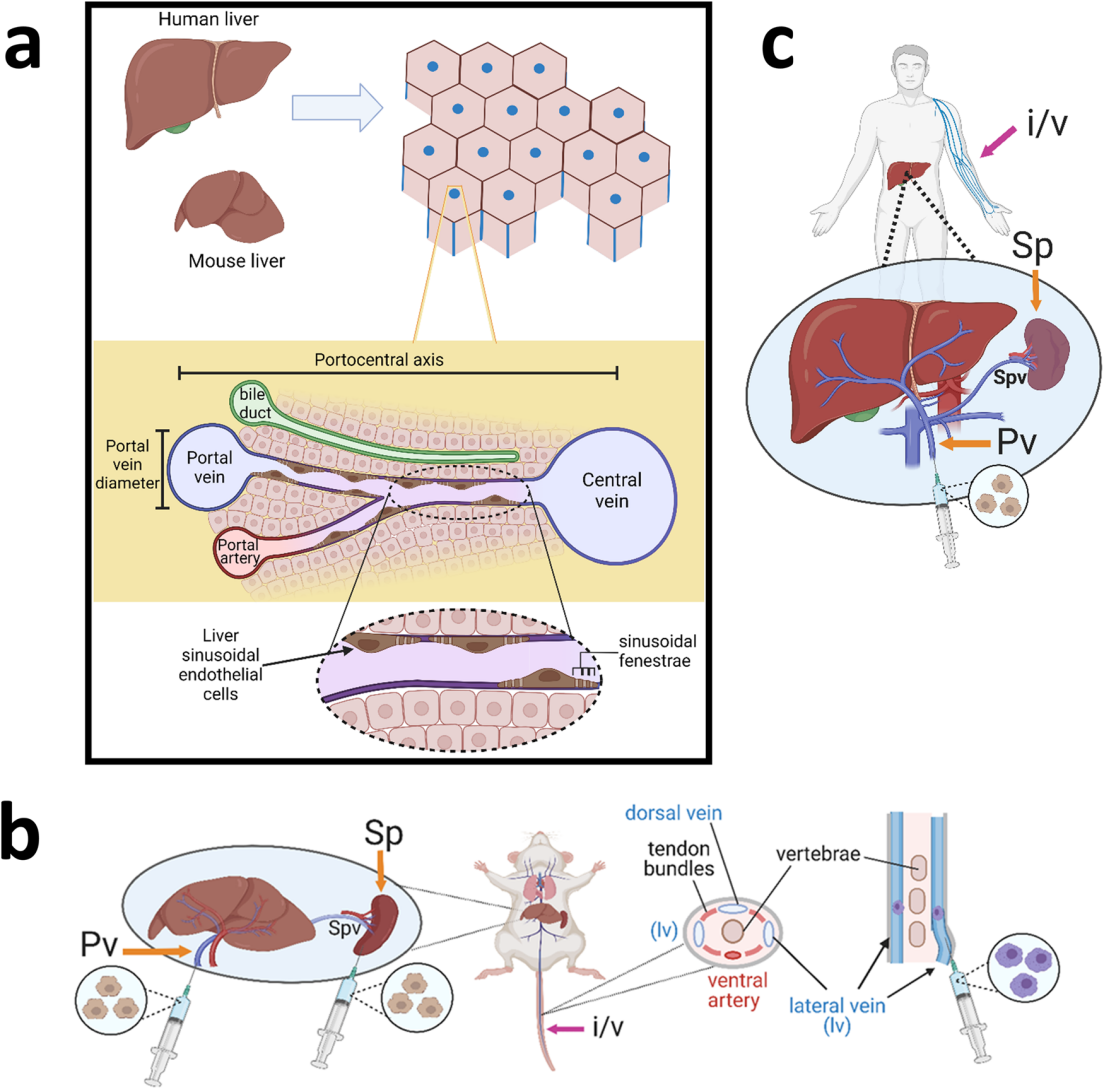
Lengthscale variations between mouse and human liver architecture. **a** Schematic showing elements of liver architecture and regions which can vary between mice and humans. Top panel: Mouse and human livers which have a repeating lobular structure. Middle panel: Cross sectional representation of a liver lobule, highlighting the portocentral axis and portal vein diameter, which are statistically different in size between mouse and humans. Lower panel: Close up of a sinusoid, highlighting the sinusoidal endothelium and associated fenestrae. **b**, **c** Schematics demonstrating differences between sites of hepatic cell therapy administration in mouse and humans. Sp spleen, Pv portal vein, Spv splenic vein, i/v intravenous, lv lateral vein. Brown polyhedral cells represent hepatocytes, purple cells represent macrophages.

Taking HTx as an example, preclinical rodent studies have established that following intrasplenic infusion, the large size of transplanted hepatocytes causes transient portal hypertension as a result of entrapment within liver sinusoids. This occlusion of blood flow activates ischaemia–reperfusion events and the recruitment of innate immune cells (including liver resident macrophages known as Kupffer cells, natural killer cells and monocytes)^32,33^. The majority of donor hepatocytes lodged in the sinusoids and portal spaces are subsequently cleared by the recruited immune cells (an estimated 70–80% of the transplanted population)^34^. This induces a release of cytokines from Kupffer cells, yielding increased vascular permeability and assisting the translocation of surviving hepatocytes across sinusoid fenestrations to enable engraftment within the liver parenchyma^34,35^. Even if the immune responses directly match between preclinical animal models and humans, there is a statistically significant difference in the diameter of mouse and human sinusoidal fenestrae, an important final juncture for the transit of surviving cells into the parenchyma^34,35^. Table 1 shows measured variations between mouse and human liver architecture with the potential to influence liver cell therapy success, including hepatocyte cell size (mean diameter) and volume^36–43^, and the distance along the hepatic sinusoids between the portal tracts and the central veins (known as the portocentral radius) (Table 1, Fig. 1)^44–46^.

**Table 1:** Variations between mouse and human hepatocytes and associated liver architecture

Liver/hepatocyte characteristic/feature	Mouse		Human
Mean cell diameter (µm)^a^	21 µm (60–75% of cells), 16 µm (5–15% of cells), 27 µm (15–25% of cells) (*n* = 6 independent experiments)		14–25 µm (*n* = 50 different batches)
Mean volume of cell (µm^3^)^b^	Mononucleated	Binucleated	3000, *n* = 7 cells
	2 populations:−3126 ± 1302 (~14% of cells) & 5313 ± 1175 (~10% of cells)	2 populations:- 5678 ± 1176 (~45% of cells) and 10606 ± 1532 (~30% of cells)	
Mean distance of portocentral axis (µm)^c^	211 (*n* = 8 mice)		385 (*N* = 2 independent livers)
Average diameter of sinusoidal fenestrae^d^	141 ± 5.4 nm, *N* = 4 livers, *n* = 530 ± 79 fenestrae measured per liver		107 ± 1.5 nm, *N* = 6 donors measured, 528 ± 142, fenestrae measured per 1cm^3^ biopsy
Portal vein diameter (*d*) and length (l)^e^	0.5–1 mm (d) and 5 mm–1 cm (l)		11–13 mm (d) and 7–8 cm (l)
Splenic vein diameter (mm)^e^	0.3–0.5		10

^a^Measurements performed on isolated cells in suspension using an electronic analysis machine (129SvJ mice) or an image cytometer (human cells, both fresh and cryopreserved)^39,40^.

^b^For mouse cells analysis performed on 2559 hepatocytes within fixed tissue, *n* = 3 mice (strain not specified)^38^. For humans analysis based on protein content calculation of mononuclear cells^36,37^.

^c^Mouse: 6 images taken and 300 fields of view analysed to produce measurements)^83^, human: image analysis of paraffin fixed tissue samples, range 171–625, *n* > 325 measurements per liver)^84^.

^d^Human biopsies obtained from the distal rim of the left lobe of donors without signs of primary or secondary liver disease undergoing elective laparascopic cholecystectomy (gall bladder removal). Diameter is statistically significantly smaller than C57BL/6 mice *p* < 0.01^44,45^.

^e^Vascular length and diameter measurements from refs. ^85,86^.

Many animal models are selectively bred to be immunosuppressed to ensure successful xenogeneic cell transplantation. Such models cannot fully recapitulate potential innate or adaptive immune responses resulting from cell transplantation. Additionally, differences in murine immunocompetencies affect the severity of the induced liver injury, and as the degree of liver injury impacts the proportion of donor cells that successfully repopulate the liver, this impacts the engraftment readout^47–50^. Immune responses are known to impact both short-term and long-term cell therapy survival, thus immune system mismatches between animal models and humans unavoidably restricts the predictive capability of preclinical models for clinical outcomes.

Finally, the site of cell administration between animal and human studies also varies. HTx in rodents is frequently delivered intrasplenically, whereas in adult human patients HTx intraportal infusion is more common^51^, either via transhepatic puncture of an intrahepatic branch of the portal vein, or by entering a small splenic vein branch (Fig. 1)^52^. This difference in delivery site between rodents and humans is largely due to technical challenges resulting from the small size of the rodent portal veins and the reduced experimental reproducibility with rodent portal delivery due to increased blood pressure backflow. In contrast, the spleen is anatomically easy to access in rodents and therapeutic cells immediately migrate to the liver parenchyma. Despite the preference for intraportal delivery in humans, this is only a suitable route of delivery for cell therapies in certain liver diseases, such as inborn errors of metabolism and ALF. Portal hypertension frequently accompanies chronic liver disease and is often cited as a contraindication for cell transplant via the portal vein. In humans the cut-off pressure at which intraportal cell transplant is contraindicated is different across cell types^5,53^. More research is required to determine the critical pressure beyond which it is unsafe to deliver HTx via the portal system in humans.

## Tracking cell delivery and engraftment

To elicit function, cell therapies must reach the correct place. Clinical indicators of transplanted cell efficacy, such as monitoring changes in serum liver enzymes, are typically used to assess therapeutic success. Cell engraftment and survival are also measured by histological analyses. In clinical studies this can be achieved by analysing repeat biopsies^54^ or collected liver tissues (following OLT where HTx has acted as a bridging therapy), and in preclinical studies tissues can be obtained from sacrificed animals at intermediate or experimental end points. Whilst these markers give valuable insight into surviving or engrafted cell functionality, they are not linked to quantitative viability data (i.e. they do not provide a directly measurable indication of cell survival relative to total input cells, for example as a 3D reconstructed image), and thus give no indication of the proportion of transplanted cells that remain viable, nor whether transplanted cells expand or become depleted over time. This is a bottleneck for directly improving initial engraftment or homing to the injured niche or comparing transplantation strategies, as, without the technology to non-invasively image donor cells post-transplantation, it is not possible to quantitatively determine whether homing or engraftment can be improved by emerging regenerative strategies such as co-administration of supporting cells and growth factors or cell delivery via different transplantation sites. Currently, there are no other methods to assess the status of the transplanted cells beyond these inferential blood/enzyme markers. These give only a global picture of the liver health of the patient, rather than specifically reporting on the transplanted cell population. Hence, without imaging, it is not possible to directly determine the whole-body fate of the cells post transplantation. Wider use of non-invasive in vivo imaging technologies to quantitatively monitor the longitudinal survival of transplanted cells along with the spatial information provided at the whole-body level can be coupled with serum analyses to pinpoint changes in cell populations over time (e.g. for HTx this would improve understanding of graft failure over time in order to better predict immunogenic responses leading to graft transience).

In vivo cell tracking allows evaluation of the whole-body distribution of administered cells (rather than imaging focused only on the site/organ of cell transplant thus informing on potential migration to unexpected sites together with the associated kinetics) and their persistence and/or expansion. Repeat imaging of the same subjects (patients or animals) over time reduces inter-subject variability compared to conventional approaches (where animal cohorts are sacrificed at different time points) and allows monitoring of changes in the transplanted cell population in real-time, ultimately improving output data. Despite these benefits, the underutilisation of in vivo imaging approaches means there is a lack of quantitative data on the viability and whole-body distribution of cell therapies in real-time post-transplant and thus a gap in understanding how this affects therapeutic outcomes.

Successful tracking strategies (discussed previously^55^) typically either modify cells to express a suitable imaging reporter gene (longitudinal studies) or use an appropriate direct cell labelling agent such as superparamagnetic particles (MNPs) or a radionuclide (short-term studies)^56,57^. In the latter approach labelling agents are introduced to cells prior to transplantation and are subsequently used for cell monitoring (in the case of MNPs via magnetic resonance imaging). However, labelling agents can decouple from administered cells, for example via cell death or phagocytosis by host macrophages, so direct labelling approaches cannot be used to assess cell viability.

Alternatively, reporter gene-based cell tracking, which relies on expression and detection of the reporter protein, ensures the imaging signal is only detected where viable therapeutic cells persist^55^. This can provide valuable insight in preclinical studies, while reducing animal numbers (and thus experimental costs) as the same animals can be repeatedly imaged at multiple timepoints of interest and cell survival quantified via image analysis following each imaging session, rather than sacrifice of separate animal cohorts at each timepoint of interest to analyse cell survival histologically. Although genetically engineering therapeutic cells may dissuade those looking for an easy path to clinical translation due to additional regulatory hurdles, this approach has already been used for a first-in-man cell therapy study tracking cytotoxic T-cell therapy in glioblastoma patients^58^. Reporter gene expression enabled the trafficking of the therapeutic cells to the tumour site within the brain to be monitored with high precision by PET/CT imaging. This positron emission tomography (PET) radionuclide imaging approach combined with computed tomography (CT) (which provides a cross-sectional 3D reconstructed x-ray), enabled potential trafficking of cells to distant tumour foci to be assessed. As gene editing becomes more readily adopted for novel cell therapies, these modifications may become more commonplace in clinical studies^59^. Ultimately, greater utilisation of in vivo imaging in experimental studies using either temporary cell labelling or reporter gene approaches is essential to enhance our understanding of the biodistribution and survival of candidate liver cell therapies. Integrating these quantitative temporal and spatial experimental datasets with in silico modelling approaches will enable validated models to be developed which will assist in identifying mechanisms to promote liver regeneration and accelerate translation of therapies to the clinic.

## Mathematical modelling to enhance translation of cell therapies

### Islet cell therapies

A clinical example of a statistical model used in the context of cell therapy is the BETA-2 score. The BETA-2 score is used to evaluate β-cell function following intraportal islet cell transplant (ITx) for patients with type 1 diabetes mellitus patients. The BETA-2 score is a non-invasive method for monitoring graft function that relies on four measurements obtained from a single fasting blood sample which are combined into a composite score of β-cell function, standardised to body weight^60^. The BETA-2 score was preceded by the β-score^61^ which assigned scores to four variables indicative of glycaemic control in ITx patients as a non-invasive means of monitoring islet graft function. Scores of 0 (clearly abnormal/in diabetic range), 1 (intermediate) or 2 (normal) were assigned to the measured values (with scores ranging from 0-8, with 8 being a perfect score). However, changes in treatment confounded the β-score due to the interdependence between variables. The BETA-2 score, developed using stepwise forward linear regression from the original β-score data set, instead incorporates continuous values of the independent variables enabling changes in ITx graft function to be more accurately and sensitively detected^60^. Variables include: fasting connecting peptide (secreted by β-cells in equimolar concentrations with insulin^62^), plasma glucose, proportion of red blood cells with glycated haemoglobin A1c (a form of glycated haemoglobin found in red blood cells that can be indicative of excessive blood sugar levels) and administered insulin dose, and as these continuous variables are standardised to body weight the score readily accommodates changes to treatment over time. BETA-2 has been independently evaluated as accurately reflecting islet graft function with high sensitivity^63^. In instances where engrafted cells remain functional, ITx can maintain patients for up to 5 years, with BETA-2 predicting graft deterioration ≥9 months *in advance* of graft failure^64^. While other simple indices to estimate β-cell function after ITx exist, a comparative study showed that the BETA-2 score was one of the two best indicators of success^65^. The BETA-2 score is an example of the type of potential output that in silico modelling could bring to candidate liver cell therapies over the long term.

### Hepatitis C virus infection with interferon treatment

**Fig. 2:**
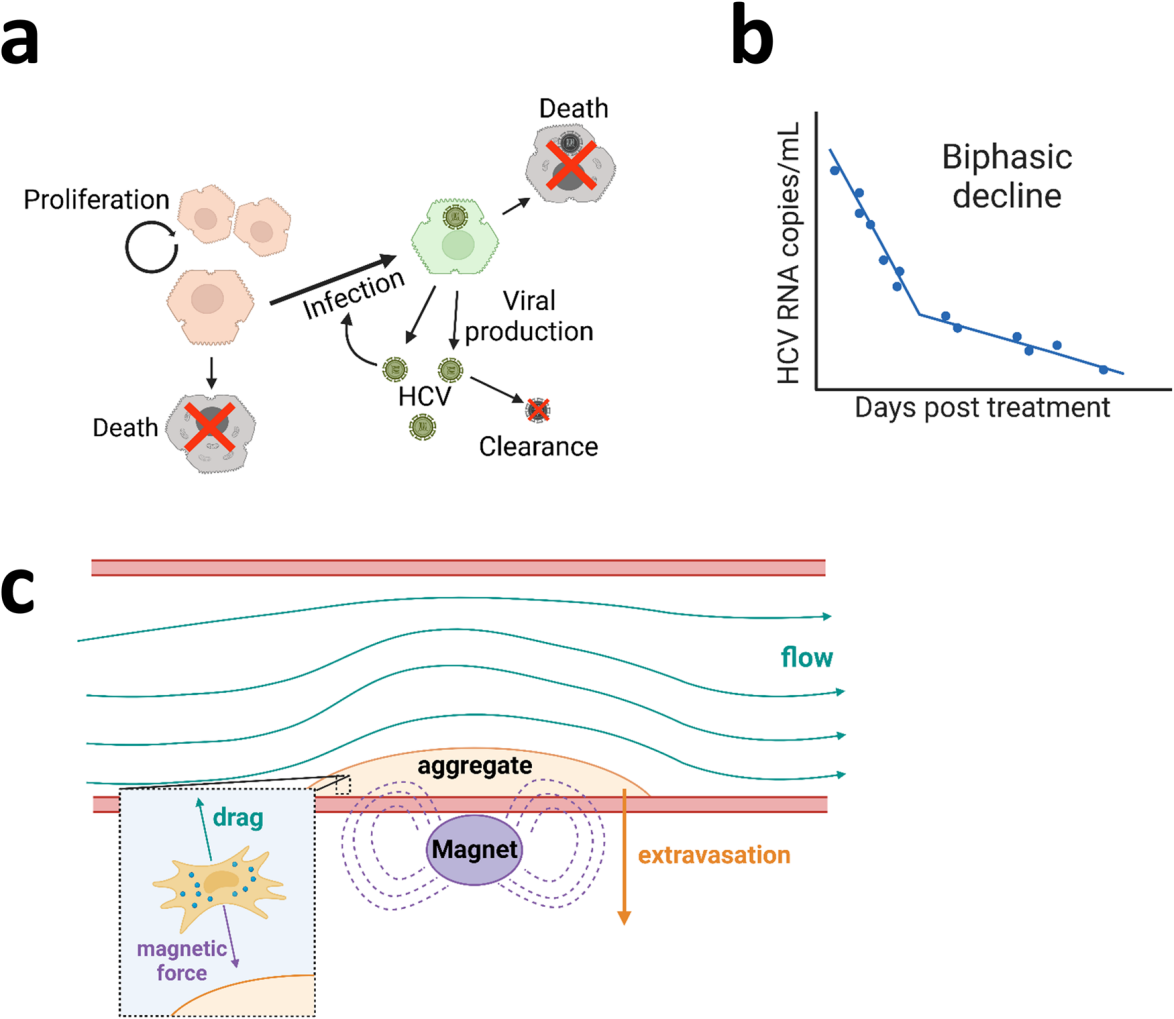
Highlighting the power of mechanistic mathematical modelling. **a** Biological schematic showing the parameterised elements of the HCV kinetic model used to generate the mechanistic equations. Infected hepatocytes (target cells), virus concentration (HCV RNA), hepatocyte number when therapy begins (assumed to be constant due to their slow turnover during homoeostasis/treatment duration), hepatocyte infection rate, and viral production rate (in the absence of treatment) were all parameterised for. By separately incorporating reduced hepatocyte infection and reduced viral production rates and using collected data on patient viral loads in the model, they elucidated that IFN-α acts by reducing the viral production rate (rather than hepatocyte infection rate). Orange polyhedral cells represent healthy hepatocytes, green polyhedral cells represent HCV-infected hepatocytes, grey polyhedral cells represent dead hepatocytes. **b** Representative graph of the biphasic HCV decline trend seen from the patient sample data during interferon treatment, which underpins the model. **c** Simplified schematic demonstrating key parameters incorporated into the mathematical model of magnetic cell targeting. Inset depicts the combined actions of magnetic force and fluid drag acting on magnetic particle-labelled cells flowing through the system.

A powerful example of the application of mechanistic modelling in hepatology arises from determining the kinetics of hepatitis C virus (HCV) infection during interferon-α (IFN-α) treatment. Prior to development of a mathematical model, the therapeutic mechanism of IFN-α was not well understood, with treatment successful in only 15–30% of cases^66^. Neumann et al. adapted a flagship mechanistic model describing the viral kinetics of HIV during antiretroviral treatment^67^ to describe the dynamics of target cells, infected cells and viral load (HCV RNA)^68^ (Fig. 2a). The authors hypothesised that IFN-α acts to either reduce the production of virions from infected cells or reduce the de novo rate of infection. By capturing these hypotheses within the mathematical model, simulating both scenarios and comparing model predictions against robustly collected data on patient viral loads following daily IFN-α treatment at different doses (blood samples collected every few hours for 2 days and then daily for 2 weeks) the authors elucidated that IFN-α acts by reducing the viral production rate (rather than hepatocyte infection rate). Their model also explained the biphasic decline of viral load seen in the experimental data: the viral load initially declines rapidly, after which the decline is more gradual (Fig. 2b). The rapid decline was hypothesised to result from quick removal of free virus, with the gradual reduction accounted for by the slower death rate of virus producing, infected hepatocytes. This ground breaking study, which has been cited in more than 2750 subsequent publications, highlights the impact mathematical modelling can have in gaining insights into biological systems underpinned by numerous complex interactions that would be exceptionally challenging to tease apart by experimental methods alone.

Multiple mechanistic models describing patient response to novel HCV treatments have subsequently been developed building on the viral dynamics from Neumann’s model, adding complexity as needed^69^. Examples include models able to capture post-treatment rebounds in viral load resulting from resistant viral strains^70^, and in silico studies that predict that combination therapy approaches would prevent resistance in most patients, enabling sustained virological response and reduced treatment duration^71^. These latter predictions were later confirmed in clinical trials^72^. These examples demonstrate the pivotal role mechanistic modelling can play in advancing our understanding of the biological response to treatments, and how this understanding can identify novel therapies that can advance towards the clinic.

### Magnetic stem cell targeting

Finally, we showcase an example of mechanistic mathematical modelling specifically in the field of cell therapy motivated by the growing interest in magnetic cell targeting approaches which have the potential to enhance delivery of cell therapies to injury sites. Intravenous injection consistently results in non-specific biodistribution of injected cells compared to the more invasive approach of directly delivering cells to injury sites. Therapeutic failure in human MSC trials is often attributed to insufficient homing to target sites, with pre-clinical and clinical studies demonstrating only 10% of intravenously delivered MSCs persist at targets after injection^73^. Magnetically labelling cells with MNPs prior to administration allows control of cell delivery to, and retention at, target sites using anatomically directed magnetic fields^74^. The magnetic nature of the label also opens up the possibility for tracking tagged cells using non-invasive imaging methods such as MRI. Magnetically-aided cell delivery is also used in cancer therapies. For example, in the context of liver cancer, microbots carrying stem cells are injected into the portal vein. The microbots are then actuated by the application of a magnetic field causing them to release their stem cell cargo which goes on to greatly inhibit tumour growth^75^. Magnetic nanoparticles also underpin many drug delivery systems, see, for example Aslam et al. and references therein^76^.

Translating targeting approaches to the clinic requires identifying the optimal conditions for therapeutic efficacy, such as: concentration of magnetic labelling agent for cell internalisation, required strength and duration of imposed magnetic field to ensure cell delivery/retention at target site, and the number of injected cells needed for efficacy. Safety challenges such as concentration and infusion rate of magnetically tagged cells to avoid cell aggregates and obstruction of vessels close to the injury site must also be considered.

To address these questions, Yeo et al. developed a mathematical model based on magnetically-tagged MSCs flowing through a channel filled with plasma containing red blood cells (RBCs) subject to a magnetic field due to a magnet located at the lower boundary^77^. Experimental data obtained from in vitro models of this system shows MSC trapping increases with increased MSC magnetic load and decreases with higher RBC concentration^78^. Cells mobilise through the channel due to drag from the surrounding viscous fluid, induced magnetic velocity and collisions with RBC resulting in a diffusive-motion of the MSCs. Magnetically captured MSCs were assumed to form a solid aggregate on the wall of the channel near the magnet. Erosion of the aggregated cells was included proportional to the shear stress exerted by the flowing fluid on the aggregate surface. The model was also extended to account for the in vivo scenario where MSCs extravasate across the vessel wall towards the site of injury. This latter feature is not captured in the in vitro models and shows how mathematical models can be used to bridge the gap between in vitro experimental data and the in vivo scenario.

The mathematical model was validated by comparing predictions with the in vitro data, and the model was utilised to provide insights into the dynamics of cell capture and the impact of MSC magnetic load and magnetic field strength on the extent of aggregate build up. The theoretical modelling predictions of the in vivo outcome due to extravasation remain to be tested in animal models. Furthermore, the mechanistic mathematical modelling enables rapid exploration of the role of parameters such as vessel size, cell number, magnetic field strength on the safety and efficacy of the therapeutic intervention which would be prohibitive to explore experimentally. This study highlights how bridging the gap between in vitro and in vivo or preclinical and clinical studies with appropriate modelling could accelerate the advance of promising cell therapies and novel approaches to clinical translation.

### Exploiting the power of mathematics to advance cell therapies

We argue that harnessing the potential of in silico modelling approaches will play an equally important role in overcoming translational bottlenecks for liver cell therapies. These bottlenecks include: ensuring the safe and effective delivery of the cells to the disease site; preventing an immunogenic response; and ensuring successful cell engraftment and/or function at the injury site. Relevant mechanistic models for each of these bottlenecks have been developed which could be exploited to enhance liver cell therapy with appropriate adaptation and supporting experimental data sets^79–81^. For example, during transit through the vasculature, from injection site to injury site, transplanted cells experience biomechanical cues such as fluid shear and pressure. These cues are key as they modulate cell phenotype, function and epigenetic fingerprints. Theoretical fluid mechanics models, in which individual cells or populations of cells are considered, can be employed to assess the local biomechanical environment experienced by transiting cells and thereby predict the fraction of injected cells that reach the injury site^77^. Encapsulating cells in biodegradable scaffolds prior to injection can modulate the mechanical cues experienced by donor cells transiting to the injury site, as well as enhance their subsequent engraftment within the injured tissue niche^79^, and mechanistic mathematical models that account for the interplay between the cell, the encapsulating material (which may be poroelastic), and surrounding fluid can predict the modulated biomechanical cues experienced by the cell. Mathematical models that incorporate subcellular features, such as the actin-myosin network within the cytosol^80^, enable detailed consideration of the impact of the stress experienced by the cell membrane on the intracellular stress distribution, an important feature when considering mechanostransduction mechanisms. Finally, when considering the interplay between the donor cells and the injured tissue niche, a natural first step is to develop differential equation models describing the interplay between the donor and host cells, together with the extracellular matrix environment, see ref. ^81^ for an example of this approach used to capture the interplay between cytotoxic and helper T cells in a tumour microenvironment. All these types of quantitative mathematical models can be used to improve protocols, for example by determining optimal cell numbers and delivery routes, or by generating experimentally testable hypotheses leading to new understanding that can be exploited to develop next-generation cell therapies. Finally, mathematical models can compensate for the inherent differences between animal model systems and humans (Table 1), thereby translating promising preclinical strategies into clinical scenarios.

## Discussion

Mathematical modelling is a powerful tool to advance cell therapy translation that has been underutilised for liver cell therapies. Mechanistic models are uniquely positioned to benefit the field, by stimulating progress even where data is limited or difficult to generate. Using in silico models to account for differences between preclinical models, humans and/or different types of transplanted cell populations could significantly reduce preclinical costs during novel therapy development, enhance the efficacy of current regimens, and accelerate translation. For mechanistic mathematical models to accurately capture the key biological processes it is essential that they are informed by quantitative experimental data, for example, the degree of cell survival post-transplant (particularly longitudinally), and the transplanted cell spatiotemporal kinetics. We advocate for greater incorporation of appropriate non-invasive in vivo imaging strategies in transplantation studies to aid in silico model development. We have showcased how mechanistic models played a critical role in understanding early HCV drug treatments and the translational development of subsequent therapeutic regimens in that arena. Given the European Parliament resolution passed in 2021 calling on the European Commission to phase out all animal experiments^82^ it is now timely for mechanistic models, in combination with non-invasive imaging tools, to contribute to reducing the number of animal experiments and act at the forefront of initiatives advancing next-generation therapies for liver diseases.

### Reporting summary

Further information on research design is available in the Nature Research Reporting Summary linked to this article.

### Supplementary information

Reporting summary
